# Supplementary material for: Metabolic regulation of pluripotency and germ cell fate through α‐ketoglutarate
Source: EMBO J. 2018 Sep 26;38(1):e99518. doi: 10.15252/embj.201899518 (PMC6315289; doi:10.15252/embj.201899518)
Supplement: Supplementary file 1 — Appendix [file EMBJ-38-e99518-s001.pdf]

Appendix

Table of contents

Appendix Figure S1. OSN binding site analysis.

Appendix Table S1. KL-divergences for metabolic regulators.

Appendix Table S2. Genes for fitting the pseudotime model.

Appendix Table S3. P-Values Table.

Appendix Figure S1

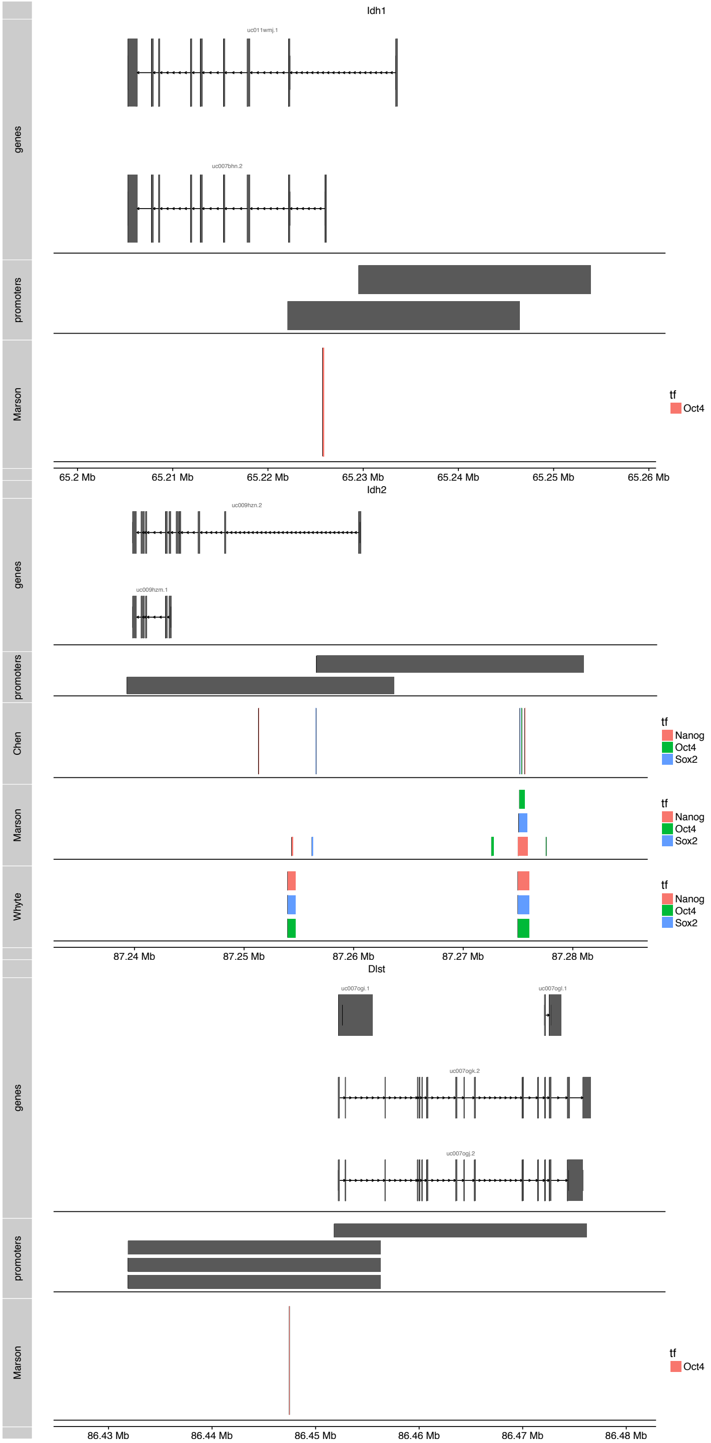

Appendix Figure S1. OSN binding site analysis.
Graphical representation of OCT4, SOX2, and NANOG (OSN) occupancy within 20Kb upstream to 4Kb downstream of the transcriptional start sites of *Idh1*, *Idh2*, and *Dlst*, respectively, based on three published ChIP-seq data sets (Chen, Xu et al., 2008, Marson, Levine et al., 2008, Whyte, Orlando et al., 2013).

Gene	KL	Name
NM_023755	136.5236308	*Tfcp2l1*
NM_021480	66.1180974	*Tdh*
NM_053079	64.78301425	*Slc15a1*
NM_001159500	59.24311428	*Esrrb*
NM_010637	45.16484143	*Klf4*
NM_001040400	30.737241	*Tet2*
NM_011978	26.87833371	*Slc27a2*
NM_010203	25.80136211	*Fgf5*
NM_001099276	25.54584239	*Pik3c2b*
NM_001003960	23.79742667	*Dnmt3b*
NM_008183	17.0763945	*Gstm2*
NM_001253679	14.76349961	*Slc7a7*
NM_009944	14.68719555	*Cox7a1*
NM_001160180	13.91573125	*Tor1aip2*
NM_028016	13.5537245	*Nanog*
NM_001003961	12.17205131	*Dnmt3b*
NM_013495	11.77658891	*Cpt1a*
NM_001122997	10.66290782	*Dnmt3b*
NM_001038695	9.957209816	*Kdm3a*
NM_008960	9.851206911	*Pten*
NM_001160181	9.630911571	*Tor1aip2*
NM_175935	9.366872286	*G6pc3*
NM_173011	9.241755624	*Idh2*
NM_172436	8.425208893	*Slc25a12*
NM_001136069	8.306550629	*Ldha*
NM_027439	8.066051293	*Atp6ap2*
NM_007747	8.03619448	*Cox5a*
NM_001031772	7.876657316	*Lin28b*
NM_008492	7.322423777	*Ldhb*
NM_010699	7.29780368	*Ldha*
NM_001146100	6.962250648	*Hk1*
NM_172665	6.953297813	*Pdk1*
NM_145833	6.743466644	*Lin28a*
NM_172843	6.597114656	*Tor1aip2*
NM_001081182	6.162208023	*Atp8b2*
NM_001172095	5.89819671	*Kdm4c*
NM_013671	5.828072212	*Sod2*
NM_011492	5.425501274	*Stk11*
NM_008709	5.275073616	*Mycn*
NM_001111320	5.214763785	*Idh1*
NM_009415	5.108287653	*Tpi1*
NM_009716	4.901152355	*Atf4*
NM_025628	3.931267757	*Cox6b1*
NM_001242396	3.923271459	*Jmjd1c*
NM_015731	3.897092538	*Atp9a*

NM_011400	3.568088135	*Slc2a1*
NM_001111288	3.399598705	*Sco2*
NM_175094	3.386092857	*Pdhx*
NM_010359	3.332339942	*Gstm3*
NM_019739	3.28267624	*Foxo1*
NM_019703	3.154870194	*Pfkp*
NM_183220	3.112213109	*Accs*
NM_001081256	3.034419975	*Kdm3b*
NM_133826	2.999471784	*Atp6v1h*
NM_025973	2.890706328	*Pgc*
NM_011443	2.885444269	*Sox2*
NM_008839	2.865849187	*Pik3ca*
NM_030225	2.849991135	*Dlst*
NM_001177307	2.803183195	*Aldoa*
NM_011401	2.777608238	*Slc2a3*
NM_018789	2.74675669	*Foxo4*
NM_025494	2.664283917	*Atp6v1c1*
NM_010324	2.648100212	*Got1*
NM_007510	2.473248138	*Atp6v1e1*
NM_019488	2.371505725	*Slc2a8*
NM_001109757	2.334284827	*Atp7a*
NM_010497	2.298536711	*Idh1*
NM_011032	2.21817507	*P4hb*
NM_080467	2.160140668	*Atp6v0a4*
NM_024200	2.140132576	*Mfn1*
NM_008826	2.079573308	*Pfkl*
NM_008133	2.01681379	*Glud1*
NM_016774	1.988253774	*Atp5b*
NM_008841	1.905943488	*Pik3r2*
NM_207215	1.875156746	*Mycbp2*
NM_181586	1.867105151	*Sirt6*
NM_011405	1.86210747	*Slc7a7*
NM_001159589	1.857653113	*Sirt1*
NM_009721	1.855012206	*Atp1b1*
NM_001029837	1.842031216	*Pik3cd*
NM_010513	1.682383127	*Igf1r*
NM_001163430	1.549433937	*Sirt6*
NM_183138	1.530409498	*Tet3*
NM_133667	1.490495189	*Pdk2*
NM_001161712	1.490048373	*Gcat*
NM_009662	1.470809205	*Alox5*
NM_008810	1.461813203	*Pdha1*
NM_001037863	1.453721013	*Atp11c*
NM_019660	1.452958226	*Mycbp*
NM_007505	1.443388258	*Atp5a1*
NM_172943	1.401109846	*Alkbh5*

NM_009804	1.383737885	*Cat*
NM_007438	1.370041743	*Aldoa*
NM_133249	1.356469527	*Ppargc1b*
NM_198027	1.348694819	*Alkbh6*
NM_144787	1.331494177	*Kdm4c*
NM_001012518	1.322151752	*Ehmt1*
NM_010924	1.320716894	*Nnmt*
NM_001112738	1.312611249	*Atp5c1*
NM_013910	1.298427918	*Kdm2b*
NM_001205339	1.28434913	*Psat1*
NM_009660	1.276535144	*Alox15*
NM_145981	1.268300886	*Phyhip*
NM_010568	1.260367505	*Insr*
NM_011434	1.227102443	*Sod1*
NM_001036684	1.217154553	*Atp2b2*
NM_133198	1.176979173	*Pygl*
NM_175015	1.168146253	*Atp5g3*
NM_013415	1.16428202	*Atp1b2*
NM_026444	1.15907378	*Cs*
NM_173001	1.158194983	*Kdm3a*
NM_001037937	1.153883032	*Deptor*
NM_178386	1.15325574	*Slc25a31*
NM_016755	1.09339947	*Atp5j*
NM_008618	1.079111938	*Mdh1*
NM_138679	1.071824995	*Ash1l*
NM_025910	1.059373031	*Riox2*
NM_026922	1.057047261	*Atp2c2*
NM_145494	1.029894462	*Me2*
NM_145630	1.029246657	*Pdk3*
NM_028386	1.02768843	*Asphd2*
NM_007994	1.016506074	*Fbp2*
NM_007861	1.016370289	*Dld*
NM_001033310	1.010569268	*Cox18*
NM_001163641	0.988796846	*Setdb1*
NM_010292	0.976599081	*Gck*
NM_029573	0.960778946	*Idh3a*
NM_145470	0.954952729	*Deptor*
NM_080633	0.936684633	*Aco2*
NM_001198933	0.928565155	*Me1*
NM_008173	0.925604891	*Nr3c1*
NM_001193271	0.920817407	*Meis1*
NM_009723	0.911506969	*Atp2b2*
NM_013477	0.901767755	*Atp6v0d1*
NM_145569	0.886938415	*Mat2a*
NM_026536	0.877160049	*Atp5s*
NM_001243053	0.873992415	*Bcat2*

NM_175460	0.854063649	*Nmnat2*
NM_011785	0.851235735	*Akt3*
NM_172132	0.842449376	*Kdm4b*
NM_133872	0.839972271	*Kdm1a*
NM_001080809	0.837695878	*Cps1*
NM_020009	0.831999334	*Mtor*
NM_001127233	0.831450019	*Trp53*
NM_001081212	0.829382931	*Irs2*
NM_001199296	0.821229493	*Acly*
NM_001163642	0.811409348	*Setdb1*
NM_026094	0.795893472	*Atp8b3*
NM_001039645	0.773051599	*Asphd1*
NM_181040	0.754084431	*Atpaf1*
NM_022724	0.750942258	*Suv39h2*
NM_007750	0.73373021	*Cox8a*
NM_175111	0.725806687	*Hspbap1*
NM_021877	0.720552442	*Hr*
NM_001081081	0.705210135	*Gls*
NM_007512	0.703754649	*Atpif1*
NM_019740	0.667227361	*Foxo3*
NM_025321	0.663332217	*Sdhc*
NM_007749	0.646908151	*Cox7c*
NM_008615	0.642949004	*Me1*
NM_197980	0.64103693	*Cox19*
NM_144533	0.629492888	*Nmnat3*
NM_011528	0.624342848	*Taldo1*
NM_001243052	0.609472729	*Bcat2*
NM_138758	0.604916123	*Tmlhe*
NM_130451	0.603969868	*Slc2a10*
NM_133435	0.60182131	*Nmnat1*
NM_011640	0.59795626	*Trp53*
NM_007509	0.5950886	*Atp6v1b2*
NM_153056	0.594640166	*Sirt7*
NM_001001984	0.586491797	*Kdm2a*
NM_009887	0.583144847	*Cer1*
NM_001252288	0.578964079	*Ogdh*
NM_178029	0.577833801	*Setd1a*
NM_009725	0.577004099	*Atp5f1*
NM_025461	0.570272145	*Cox16*
NM_001177753	0.563132694	*Pfkfb3*
NM_033325	0.553446387	*Loxl2*
NM_001253680	0.549403531	*Slc7a7*
NM_011786	0.54857379	*Aloxe3*
NM_033398	0.548175476	*Jmjd6*
NM_010726	0.543911265	*Phyh*
NM_011506	0.538166886	*Sucla2*

NM_007531	0.532941456	*Phb2*
NM_026944	0.513243562	*Alkbh3*
NM_172659	0.508291198	*Slc2a6*
NM_001167691	0.502714564	*Sirt4*
NM_001243051	0.501960996	*Atp6v0a1*
NM_001177627	0.486516699	*Slc2a6*
NM_007748	0.478069468	*Cox6a1*
NM_009726	0.475664388	*Atp7a*
NM_011722	0.474725924	*Dctn6*
NM_001163456	0.470273021	*Cox18*
NM_177161	0.46606486	*P4ha3*
NM_025379	0.465887808	*Cox7b*
NM_011596	0.465223111	*Atp6v0a2*
NM_008617	0.443065082	*Mdh2*
NM_013534	0.432736208	*P3h3*
NM_080793	0.418037052	*Setd7*
NM_001099779	0.41741604	*Pklr*
NM_023179	0.414136688	*Atp6v1g2*
NM_026672	0.407767064	*Gstm7*
NM_133201	0.402406423	*Mfn2*
NM_013847	0.401400792	*Gcat*
NM_178848	0.400924451	*Sirt5*
NM_173866	0.400601968	*Gpt2*
NM_009652	0.395357122	*Akt1*
NM_029094	0.391494226	*Pik3cb*
NM_001017429	0.388553578	*Cox17*
NM_018731	0.386793013	*Atp4a*
NM_145427	0.382813539	*Atpaf2*
NM_025313	0.381322244	*Atp5d*
NM_001165894	0.377657907	*Akt1*
NM_138597	0.370856903	*Atp5o*
NM_173433	0.368847459	*Kdm4d*
NM_001252287	0.362196583	*Ogdh*
NM_023633	0.35270362	*Riox1*
NM_144900	0.350672368	*Atp1a1*
NM_010942	0.348644155	*Nsg1*
NM_178934	0.345020686	*Slc2a12*
NM_007933	0.342925847	*Eno3*
NM_001256380	0.338844329	*Prdm2*
NM_007988	0.333030002	*Fasn*
NM_028133	0.328713444	*Egln3*
NM_001177308	0.326130776	*Aldoa*
NM_009659	0.321712107	*Alox12b*
NM_009737	0.320738821	*Bcat2*
NM_001160182	0.320088603	*Tor1aip2*
NM_011083	0.315420277	*Pik3c2a*

NM_177767	0.313646302	*Ogfod1*
NM_001177804	0.298267847	*Sirt3*
NM_011962	0.292734506	*Plod3*
NM_001122765	0.288246562	*Sirt2*
NM_001024955	0.287701614	*Pik3r1*
NM_008811	0.282581731	*Pdha2*
NM_010360	0.282150159	*Gstm5*
NM_011122	0.281175023	*Plod1*
NM_172382	0.276647788	*Kdm4a*
NM_053091	0.276526004	*Cox4i2*
NM_001164051	0.276031367	*Pik3cd*
NM_053207	0.275658318	*Egln1*
NM_010828	0.270546792	*Cited2*
NM_019811	0.269087266	*Acss2*
NM_001243049	0.26885761	*Atp6v0a1*
NM_009484	0.265431107	*Uty*
NM_025983	0.261812948	*Atp5e*
NM_001025250	0.260856537	*Vegfa*
NM_001077495	0.258774581	*Pik3r1*
NM_031248	0.258041963	*Lamtor2*
NM_001199274	0.257493649	*Mat2b*
NM_001102565	0.244080609	*Alkbh1*
NM_133224	0.243142144	*Atp13a1*
NM_013734	0.239511716	*Atp1a4*
NM_001177855	0.239101397	*Asph*
NM_011145	0.23868989	*Ppard*
NM_009657	0.238250671	*Aldoc*
NM_001039049	0.237349248	*Cox8c*
NM_023374	0.231524435	*Sdhb*
NM_001253857	0.227302932	*Tet1*
NM_001142804	0.225826216	*Acss3*
NM_001253920	0.222989439	*Akt1s1*
NM_009877	0.221289711	*Cdkn2a*
NM_181414	0.216966467	*Pik3c3*
NM_153389	0.215245681	*Atp10d*
NM_001177856	0.211075465	*Asph*
NM_001109686	0.208408781	*Ehmt1*
NM_009949	0.204367651	*Cpt2*
NM_010358	0.202889855	*Gstm1*
NM_001136062	0.200866143	*Eno3*
NM_001136076	0.200203224	*P4ha2*
NM_009728	0.190741059	*Atp10a*
NM_009941	0.190027952	*Cox4i1*
NM_177420	0.189603465	*Psat1*
NM_001042411	0.187689788	*P3h1*
NM_009942	0.187678502	*Cox5b*

NM_030052	0.186865668	*Cox7b2*
NM_001163487	0.18671099	*Pfkm*
NM_001167949	0.183134337	*Atp2b4*
NM_001205043	0.183101374	*Jarid2*
NM_011936	0.181299626	*Fto*
NM_025504	0.177640903	*Dmac2*
NM_029097	0.175717446	*Atp13a2*
NM_207221	0.174501037	*Jmjd1c*
NM_001114637	0.173831981	*Jmjd7*
NM_001177850	0.17106047	*Asph*
NM_001128094	0.16337336	*Atp13a3*
NM_001110266	0.153540566	*Vegfa*
NM_027188	0.153412809	*Smyd3*
NM_134017	0.153313594	*Mat2b*
NM_001033264	0.152094633	*Gls2*
NM_001253834	0.151746805	*Atp2c1*
NM_001110140	0.146903055	*Atp2a2*
NM_020582	0.146761137	*Atp5j2*
NM_001001488	0.143888803	*Atp8b1*
NM_147151	0.143328134	*Ehmt2*
NM_025576	0.141680581	*Ptpmt1*
NM_008904	0.139271683	*Ppargc1a*
NM_007386	0.138650718	*Aco1*
NM_008155	0.137852277	*Gpi1*
NM_178143	0.137024968	*Prkaa2*
NM_008131	0.136965646	*Glul*
NM_018794	0.134712188	*Atp6ap1*
NM_022433	0.131368948	*Sirt3*
NM_013743	0.130331789	*Pdk4*
NM_023326	0.130109322	*Bmyc*
NM_138595	0.129149837	*Gldc*
NM_001142916	0.127845695	*Plod2*
NM_053208	0.12775186	*Egln2*
NM_025848	0.127376307	*Sdhd*
NM_010431	0.12716126	*Hif1a*
NM_146236	0.126819496	*Tceal1*
NM_001080944	0.126252	*Atp8b4*
NM_001001798	0.125558844	*Atp11c*
NM_021878	0.121086145	*Jarid2*
NM_023281	0.118753486	*Sdha*
NM_001177752	0.11555919	*Pfkfb3*
NM_018870	0.114736262	*Pgam2*
NM_019782	0.113630105	*P3h1*
NM_011514	0.112887391	*Suv39h1*
NM_001243050	0.111273922	*Atp6v0a1*
NM_009663	0.109986151	*Alox5ap*

NM_001093757	0.109678539	*Ogfod1*
NM_028944	0.109307171	*P4htm*
NM_001127351	0.108648573	*Sirt3*
NM_182805	0.108528809	*Gpt*
NM_173371	0.108497869	*H6pd*
NM_145830	0.105270852	*Ehmt2*
NM_001164050	0.104131147	*Pik3cd*
NM_011044	0.102157621	*Pck1*
NM_009388	0.099567604	*Tkt*
NM_010209	0.098770119	*Fh1*
NM_145997	0.098537747	*Kdm5a*
NM_013820	0.098095328	*Hk2*
NM_001199177	0.097429271	*Opa1*
NM_001252470	0.096268051	*Cpt1c*
NM_183405	0.096103213	*Cox6b2*
NM_053071	0.094717158	*Cox6c*
NM_029570	0.094343255	*Atp11b*
NM_027570	0.091406642	*Ldhd*
NM_026000	0.089602472	*Psmd9*
NM_011031	0.088252185	*P4ha2*
NM_033617	0.087940349	*Atp6v0b*
NM_001081309	0.087816449	*Pik3r4*
NM_173379	0.087551705	*P3h2*
NM_028101	0.085262396	*Jmjd8*
NM_172262	0.085005168	*Kdm1b*
NM_007507	0.083663574	*Atp5k*
NM_133900	0.082745261	*Psph*
NM_152895	0.08006383	*Kdm5b*
NM_001081355	0.079312961	*Prdm2*
NM_001253836	0.079253215	*Atp2c1*
NM_008825	0.078714295	*Pfkfb2*
NM_019395	0.071598257	*Fbp1*
NM_001113354	0.071143216	*Phf8*
NM_001162416	0.068446227	*Pfkfb2*
NM_025272	0.068320212	*Atp6v0e*
NM_178405	0.066947071	*Atp1a2*
NM_177320	0.066800325	*Pik3r5*
NM_178659	0.060253203	*Jmjd4*
NM_177195	0.057848243	*Atp8b5*
NM_009727	0.057379611	*Atp8a1*
NM_001164366	0.057089022	*Atp13a2*
NM_001253831	0.055422969	*Atp2c1*
NM_011086	0.053937197	*Pikfyve*
NM_010438	0.053439074	*Hk1*
NM_001163336	0.052775601	*Atp2a3*
NM_001081274	0.050975243	*Pgd*

NM_007970	0.050930248	*Ezh1*
NM_007508	0.050749122	*Atp6v1a*
NM_178379	0.049468533	*Cox10*
NM_019812	0.049309924	*Sirt1*
NM_010729	0.048582032	*Loxl1*
NM_009875	0.04794159	*Cdkn1b*
NM_015804	0.04698102	*Atp11a*
NM_183406	0.0467239	*Cox6b2*
NM_181407	0.045399221	*Me3*
NM_177201	0.045017717	*Phf8*
NM_145614	0.044923782	*Dlat*
NM_001205181	0.043508755	*Abhd4*
NM_001160411	0.041691196	*Gstm4*
NM_001146689	0.039554403	*Ezh2*
NM_001113383	0.039209932	*Gls*
NM_019741	0.036149677	*Slc2a5*
NM_009661	0.035131152	*Alox8*
NM_001252283	0.03460966	*Ogdh*
NM_001013372	0.033531496	*Alkbh1*
NM_007971	0.033007097	*Ezh2*
NM_011078	0.032860561	*Phf2*
NM_001017426	0.031869707	*Kdm6b*
NM_011030	0.031827383	*P4ha1*
NM_028070	0.030114982	*Alkbh4*
NM_020615	0.028720209	*Atp5c1*
NM_009196	0.028710223	*Slc16a1*
NM_025396	0.028416129	*Pgls*
NM_175025	0.028084579	*Atp2c1*
NM_009722	0.027802554	*Atp2a2*
NM_023721	0.02734575	*Atp6v1d*
NM_080575	0.027125342	*Acss1*
NM_001205068	0.026305482	*Jmjd4*
NM_013580	0.025634517	*Ldhc*
NM_009948	0.025319384	*Cpt1b*
NM_013668	0.024850957	*Kdm5c*
NM_001177758	0.024645962	*Pfkfb3*
NM_025675	0.023665244	*Dph6*
NM_001031667	0.022339877	*Gsk3a*
NM_001128096	0.021277703	*Atp13a3*
NM_031376	0.021006366	*Pik3ap1*
NM_133360	0.020618454	*Acaca*
NM_153150	0.01979387	*Slc25a1*
NM_030168	0.019299166	*Rictor*
NM_001033245	0.018366633	*Hk3*
NM_001205044	0.018292888	*Jarid2*
NM_013586	0.018154656	*Loxl3*

NM_176958	0.017510417	*Hif1an*
NM_025671	0.017034066	*Ogfod2*
NM_023525	0.016962153	*Cad*
NM_181585	0.016291674	*Pik3r3*
NM_001201569	0.015731079	*Atp9b*
NM_013509	0.015728439	*Eno2*
NM_011671	0.015591862	*Ucp2*
NM_019879	0.01489249	*Suclg1*
NM_029842	0.014779935	*Kdm8*
NM_009945	0.014284486	*Cox7a2*
NM_025683	0.013931503	*Rpe*
NM_001033430	0.012285744	*Kdm7a*
NM_001252570	0.011256969	*Phyhd1*
NM_008184	0.010380485	*Gstm6*
NM_172748	0.009393959	*Fbxl19*
NM_001163488	0.00929405	*Pfkm*
NM_001003953	0.008716987	*Kdm2b*
NM_001040654	0.008307186	*Cdkn2a*
NM_133764	0.007996534	*Atp6v0e2*
NM_001177353	0.007676173	*Myc*
NM_026482	0.007392815	*Atp2b1*
NM_023058	0.006830264	*Pkmyt1*
NM_015803	0.006662922	*Atp8a2*
NM_130884	0.006355969	*Idh3b*
NM_001177849	0.006299505	*Asph*
NM_144809	0.006098807	*Prdm9*
NM_001252282	0.005976181	*Ogdh*
NM_001162946	0.005800128	*Pcx*
NM_019468	0.00579088	*G6pd2*
NM_144921	0.005717197	*NA*
NM_008323	0.00553963	*Idh3g*
NM_007502	0.005529272	*Atp1b3*
NM_144874	0.005353688	*Cox15*
NM_001164049	0.005155735	*Pik3cd*
NM_008840	0.005155735	*Pik3cd*
NM_009463	0.005155735	*Ucp1*
NM_011419	0.005026527	*Kdm5d*
NM_178149	0.004711669	*Pik3ip1*
NM_022432	0.00430147	*Sirt2*
NM_007511	0.003694542	*Atp7b*
NM_001161823	0.003511517	*Kdm4a*
NM_010241	0.003331804	*Aktip*
NM_173019	0.003186771	*Pfkfb4*
NM_199008	0.003150349	*Cox11*
NM_175016	0.002733315	*Alkbh2*
NM_001038999	0.00267217	*Atp8a1*

NM_001085529	0.002198998	*Slc2a7*
NM_010570	0.002053046	*Irs1*
NM_001177352	0.001953659	*Myc*
NM_026764	0.001863054	*Gstm4*
NM_001252568	0.001639729	*Phyhd1*
NM_007440	0.001549205	*Alox12*
NM_026270	0.001481499	*Akt1s1*
NM_022887	0.001346574	*Tsc1*
NM_134076	0.001113156	*Abhd4*
NM_011934	8.38E-04	*Esrrb*
NM_153679	8.22E-04	*Cpt1c*
NM_007504	8.05E-04	*Atp2a1*
NM_008324	7.60E-04	*Ido1*
NM_021514	7.43E-04	*Pfkm*
NM_008062	4.58E-04	*G6pdx*
NM_001162415	4.54E-04	*Pfkfb2*
NM_009483	4.44E-04	*Kdm6a*
NM_001159632	4.15E-04	*Atp6v1c2*
NM_008824	6.27E-05	*Pfkfb1*

Appendix Table S1. KL-divergences for metabolic regulators.

Listed are 478 regulators implicated in cellular metabolism, ranked by their descending KL-divergences as a quantitative measure of gene expression changes during the ESC-to-EpiLC transition. For each transcript, the RefSeq accession number (column A), the KL-divergence (column B), and the gene name (column C) are provided.

Gene	Name
NM_001253857	*Tet1*
NM_008229	*Hdac2*
NM_010264	*Nr6a1*
NM_145211	*Oas1a*
NM_001164789	*Eomes*
NM_138606	*Pim2*
NM_145630	*Pdk3*
NM_011427	*Snai1*
NM_010413	*Hdac6*
NM_001040400	*Tet2*
NM_001164148	*Tcf3*
NM_001159500	*Esrrb*
NM_172665	*Pdk1*
NM_008012	*Akr1b8*
NM_013595	*Mbd3*
NM_009337	*Tcl1*
NM_011443	*Sox2*
NM_144841	*Otx2*
NM_007883	*Dsg2*
NM_011635	*Trap1a*
NM_025788	*Nacc1*
NM_001163641	*Setdb1*
NM_010203	*Fgf5*
NM_001100186	*Zscan4d*
NM_001184709	*Tfdp2*
NM_007669	*Cdkn1a*
NM_001017426	*Kdm6b*
NM_007664	*Cdh2*
NM_145604	*D230025D16Rik*
NM_007430	*Nr0b1*
NM_008235	*Hes1*
NM_009483	*Kdm6a*
NM_023755	*Tfcp2l1*
NM_001111320	*Idh1*
NM_011263	*Rest*
NM_009769	*Klf5*
NM_010336	*Lpar1*
NM_001081209	*Prdm14*
NM_010021	*Dazl*
NM_007607	*Car4*
NM_010291	*Gjb5*
NM_009416	*Tpm2*
NM_010202	*Fgf4*
NM_021480	*Tdh*
NM_008131	*Glul*

NM_010094	*Lefty1*
NM_001031772	*Lin28b*
NM_030014	*Hook1*
NM_009522	*Wnt3a*
NM_175094	*Pdhx*
NM_007657	*Cd9*
NM_011978	*Slc27a2*
NM_025330	*Hsd17b14*
NM_001146689	*Ezh2*
NM_001033638	*Crxos1*
NM_172118	*Myl9*
NM_028615	*Dppa2*
NM_146176	*Cnot3*
NM_011852	*Oas1g*
NM_001195003	*Kat7*
NM_009846	*Cd24a*
NM_147151	*Ehmt2*
NM_001252452	*Pou5f1*
NM_001160012	*Gjb3*
NM_172887	*Fry*
NM_021273	*Ckb*
NM_018777	*Cldn6*
NM_008430	*Kcnk1*
NM_010420	*Hesx1*
NM_009211	*Smarcc1*
NM_001159769	*Nr5a2*
NM_008452	*Klf2*
NM_028053	*Tmem38b*
NM_010446	*Foxa2*
NM_028016	*Nanog*
NM_011461	*Spic*
NM_177099	*Lefty2*
NM_009538	*Plagl1*
NM_173001	*Kdm3a*
NM_144873	*Uhrf2*
NM_011618	*Tnnt1*
NM_007554	*Bmp4*
NM_021531	*Carm1*
NM_001109991	*Col18a1*
NM_008808	*Pdgfa*
NM_016689	*Aqp3*
NM_001253679	*Slc7a7*
NM_016887	*Cldn7*
NM_001081256	*Kdm3b*
NM_001037711	*Cgn*
NM_181322	*Ctcf*

NM_011588	*Trim28*
NM_001172095	*Kdm4c*
NM_010637	*Klf4*
NM_001199433	*Dnmt1*
NM_013611	*Nodal*
NM_001111079	*Uhrf1*
NM_001109686	*Ehmt1*
NM_010242	*Fut4*
NM_009801	*Car2*
NM_009758	*Bmpr1a*
NM_001081695	*Dnmt3l*
NM_001081081	*Gls*
NM_013729	*Mixl1*
NM_001136069	*Ldha*
NM_007913	*Egr1*
NM_001032378	*Pecam1*
NM_001083316	*Pdgfra*
NM_016754	*Mylpf*
NM_007477	*Arf2*
NM_173011	*Idh2*
NM_009233	*Sox1*
NM_001244200	*Pax6*
NM_001081088	*Lrp2*
NM_001177602	*Ak4*
NM_001003960	*Dnmt3b*
NM_027941	*Lrrc34*
NM_027294	*Cmtm8*
NM_172262	*Kdm1b*
NM_009196	*Slc16a1*
NM_028030	*Rbpms2*
NM_025569	*Mgst3*
NM_009864	*Cdh1*
NM_007872	*Dnmt3a*
NM_134102	*Pla1a*
NM_001033264	*Gls2*
NM_201395	*Sall4*
NM_008482	*Lamb1*
NM_008245	*Hhex*
NM_009482	*Utf1*
NM_013768	*Prmt5*
NM_011535	*Tbx3*
NM_008108	*Gdf3*
NM_010207	*Fgfr2*
NM_153791	*Flywch1*

Appendix Table S2. Genes for fitting the pseudotime model.

Listed are the 135 genes that were used to fit the pseudotime model.

For each gene, the RefSeq accession number (column A) and the name (column B) are provided.

Fig	n	*p*-value	Comparison
**2B**	2	0.000556193	+2-DG vs +dH2O
**2C**	3	0.014238593	*Lin28b* +2-DG vs +dH2O
	3	0.006765041	*EsrrB* +2-DG vs +dH2O
	2	0.046468823	*Klf4* +2-DG vs +dH2O
	2	0.010360148	*Tfcp2l1* +2-DG vs +dH2O
	3	0.030424339	*Dnmt3b* +2-DG vs +dH2O
	3	0.015651778	*Fgf5* +2-DG vs +dH2O
**2D**	2	0.042402065	+2-DG vs +dH2O
**3B**	6	1.24128E-05	+dm-αKG vs +DMSO
**3C**	5	4.67982E-06	*Tfcp2l1* +dm-αKG vs +DMSO
	5	4.00312E-05	*Klf4* +dm-αKG vs +DMSO
	5	0.000422197	*EsrrB* +dm-αKG vs +DMSO
	5	0.000600534	*Lin28b* +dm-αKG vs +DMSO
	5	8.84076E-05	*Dnmt3b* +dm-αKG vs +DMSO
	5	0.002265556	*Fgf5* +dm-αKG vs +DMSO
**3D**	4	0.028349939	+dm-αKG vs +DMSO
**3G**	2	0.047746434	+dm-αKG vs +DMSO
**3H**	2	0.153487504	*EsrrB* d2.5 +dm-αKG vs +DMSO
	2	0.032742698	*EsrrB* d5 +dm-αKG vs +DMSO
	2	0.10238989	*EsrrB* d7.5 +dm-αKG vs +DMSO
	2	0.025998096	*EsrrB* d10 +dm-αKG vs +DMSO
	2	0.004730053	*Dnmt3b* d2.5 +dm-αKG vs +DMSO
	2	0.01030498	*Dnmt3b* d5 +dm-αKG vs +DMSO
	2	0.404009	*Dnmt3b* d7.5 +dm-αKG vs +DMSO
	2	0.257992972	*Dnmt3b* d10 +dm-αKG vs +DMSO
	2	0.082621999	*Fgf5* d2.5 +dm-αKG vs +DMSO
	2	0.010375269	*Fgf5* d5 +dm-αKG vs +DMSO
	2	0.027319155	*Fgf5* d7.5 +dm-αKG vs +DMSO
	2	0.03390866	*Fgf5* d10 +dm-αKG vs +DMSO
	2	0.050105788	*Tet2* d2.5 +dm-αKG vs +DMSO
	2	0.00028121	*Tet2* d5 +dm-αKG vs +DMSO
	2	0.11442561	*Tet2* d7.5 +dm-αKG vs +DMSO
	2	0.031660152	*Tet2* d10 +dm-αKG vs +DMSO
**4A**	2	0.052568457	+dm-αKG vs +DMSO
**4B**	2	0.074290139	+BMP4 vs + dm-αKG

	2	0.052563025	+BMP4 vs +DMSO
	2	0.15674842	+dm-αKG vs +DMSO

**4C**	3	0.008864717	+dm-αKG (72h EpiLCs) vs +DMSO (72h EpiLCs)
	3	0.046092821	+dm-αKG (72h EpiLCs) vs +DMSO (48h EpiLCs)
	3	0.000276824	+DMSO (72h EpiLCs) vs +DMSO (48h EpiLCs)

**4D**	3	0.000505602	*Prdm14* DMSO_GFP+ vs DMSO_GFP-
	3	0.000594369	*Prdm14* +dm-αKG _GFP+ vs dm-αKG _GFP-
	3	0.005428973	*Prdm14* +dm-αKG _GFP+ vs DMSO_GFP-
	3	0.046649815	*Prdm14* +dm-αKG _GFP- vs DMSO_GFP-
	3	0.095377484	*Prdm14* ESCs2iLIF5%FCS vs DMSO_GFP-
	3	0.07119756	*Prdm14* +dm-αKG_GFP+ vs DMSO_GFP+
	3	0.001157328	*Tfap2c* DMSO_GFP+ vs DMSO_GFP-
	3	0.00102167	*Tfap2c* +dm-αKG_GFP+ vs +dm-αKG_GFP-
	3	0.004270274	*Tfap2c* +dm-αKG_GFP+ vs DMSO_GFP-
	3	0.00268114	*Tfap2c* +dm-αKG_GFP- vs DMSO_GFP-
	3	0.07626715	*Tfap2c* ESCs2iLIF5%FCS vs DMSO_GFP-
	3	0.072583712	*Tfap2c* +dm-αKG_GFP+ vs DMSO_GFP+
	3	0.002443949	*Prdm1* DMSO_GFP+ vs DMSO_GFP-
	3	5.74216E-05	*Prdm1* +dm-αKG_GFP+ vs +dm-αKG_GFP-
	3	0.001053135	*Prdm1* +dm-αKG_GFP+ vs DMSO_GFP-
	3	0.124352909	*Prdm1* +dm-αKG_GFP- vs DMSO_GFP-
	3	0.356249305	*Prdm1* ESCs2iLIF5%FCS vs DMSO_GFP-
	3	0.189292957	*Prdm1* +dm-αKG_GFP+ vs DMSO_GFP+
	3	9.66341E-05	*T* DMSO_GFP+ vs DMSO_GFP-
	3	0.004635653	*T* +dm-αKG_GFP+ vs +dm-αKG_GFP-
	3	0.000963986	*T* +dm-αKG_GFP+ vs DMSO_GFP-
	3	0.178289372	*T* +dm-αKG_GFP- vs DMSO_GFP-
	3	0.337346401	*T* ESCs2iLIF5%FCS vs DMSO_GFP-
	3	0.279948864	*T* +dm-αKG_GFP+ vs DMSO_GFP+
	3	0.017320005	*Klf4* DMSO_GFP+ vs DMSO_GFP-
	3	0.002457419	*Klf4* +dm-αKG_GFP+ vs +dm-αKG_GFP-
	3	0.007728492	*Klf4* +dm-αKG_GFP+ vs DMSO_GFP-
	3	0.480714886	*Klf4* +dm-αKG_GFP- vs DMSO_GFP-
	3	0.016079347	*Klf4* ESCs2iLIF5%FCS vs DMSO_GFP-
	3	0.071818665	*Klf4* +dm-αKG_GFP+ vs DMSO_GFP+
	3	0.007647435	*Tet1* DMSO_GFP+ vs DMSO_GFP-
	3	0.020882404	*Tet1* +dm-αKG_GFP+ vs +dm-αKG_GFP-
	3	0.015523272	*Tet1* +dm-αKG_GFP+ vs DMSO_GFP-
	3	0.024173901	*Tet1* +dm-αKG_GFP- vs DMSO_GFP-
	3	0.014522612	*Tet1* ESCs2iLIF5%FCS vs DMSO_GFP-
	3	0.297380564	*Tet1* +dm-αKG_GFP+ vs DMSO_GFP+
	3	0.012565123	*Kdm3a* DMSO_GFP+ vs DMSO_GFP-
	3	0.005166908	*Kdm3a* +dm-αKG_GFP+ vs +dm-αKG_GFP-
	3	0.023953856	*Kdm3a* +dm-αKG_GFP+ vs DMSO_GFP-

	3	0.00027185	*Kdm3a* +dm-αKG_GFP- vs DMSO_GFP-
	3	0.044002447	*Kdm3a* ESCs2iLIF5%FCS vs DMSO_GFP-
	3	0.036463312	*Kdm3a* +dm-αKG_GFP+ vs DMSO_GFP+
	3	0.001949632	*Kdm3b* DMSO_GFP+ vs DMSO_GFP-
	3	0.00064076	*Kdm3b* +dm-αKG_GFP+ vs +dm-αKG_GFP-
	3	0.003405594	*Kdm3b* +dm-αKG_GFP+ vs DMSO_GFP-
	3	0.017466334	*Kdm3b* +dm-αKG_GFP- vs DMSO_GFP-
	3	0.019062979	*Kdm3b* ESCs2iLIF5%FCS vs DMSO_GFP-
	3	0.023321596	*Kdm3b* +dm-αKG_GFP+ vs DMSO_GFP+
	3	0.033606632	*Gata6* DMSO_GFP+ vs DMSO_GFP-
	3	0.000891054	*Gata6* +dm-αKG_GFP+ vs +dm-αKG_GFP-
	3	0.003758573	*Gata6* +dm-αKG_GFP+ vs DMSO_GFP-
	3	0.215129728	*Gata6* +dm-αKG_GFP- vs DMSO_GFP-
	3	0.001590893	*Gata6* ESCs2iLIF5%FCS vs DMSO_GFP-
	3	0.014667648	*Gata6* +dm-αKG_GFP+ vs DMSO_GFP+

**4F**	3	0.000284149	*EsrrB* enh H3K9me2 ESCs2iLIF vs 48hEpiLCs +DMSO
	3	0.010957391	*EsrrB* enh H3K27me3 ESCs2iLIF vs 48hEpiLCs +DMSO
	3	0.007174212	*EsrrB* enh H3K9me2 48hEpiLCs +4mM dm-αKG vs 48hEpiLCs +DMSO
	3	0.005880579	*EsrrB* enh H3K27me3 48hEpiLCs +4mM dm-αKG vs 48hEpiLCs +DMSO
	3	0.000991393	*Arid5b* enh H3K9me2 ESCs2iLIF vs 48hEpiLCs +DMSO
	3	0.069955607	*Arid5b* enh H3K27me3 ESCs2iLIF vs 48hEpiLCs +DMSO
	3	0.005480763	*Arid5b* enh H3K9me2 48hEpiLCs +4mM dm-αKG vs 48hEpiLCs +DMSO
	3	0.021563945	*Arid5b* enh H3K27me3 48hEpiLCs +4mM dm-αKG vs 48hEpiLCs +DMSO
	3	0.004390116	*Tfap2c* enh H3K9me2 ESCs2iLIF vs 48hEpiLCs +DMSO
	3	0.436001715	*Tfap2c* enh H3K27me3 ESCs2iLIF vs 48hEpiLCs +DMSO
	3	0.097645967	*Tfap2c* enh H3K9me2 48hEpiLCs +4mM dm-αKG vs 48hEpiLCs +DMSO
	3	0.153514399	*Tfap2c* enh H3K27me3 48hEpiLCs +4mM dm-αKG vs 48hEpiLCs +DMSO

**EV2A**	2	0.041387046	ESCs2iLIF vs 72hEpiLCs +dH2O
	2	0.010387443	72hEpiLCs +2-DG vs 72hEpiLCs +dH2O

**EV2D**	2	0.183620097	+5mM DCA vs +dH2O
	2	0.150459221	+10mM DCA vs +dH2O
	2	0.04029871	+20mM DCA vs +dH2O

**EV2F**	2	0.157774105	*Lin28b* +5mM DCA vs +dH2O
	2	0.111931933	*Lin28b* +10mM DCA vs +dH2O
	2	0.145646414	*Lin28b* +20mM DCA vs +dH2O
	2	0.427318149	*EsrrB* +5mM DCA vs +dH2O
	2	0.21234911	*EsrrB* +10mM DCA vs +dH2O
	2	0.074394245	*EsrrB* +20mM DCA vs +dH2O
	2	0.134051939	*Klf4* +5mM DCA vs +dH2O
	2	0.112355864	*Klf4* +10mM DCA vs +dH2O
	2	0.080622567	*Klf4* +20mM DCA vs +dH2O
	2	0.349661032	*Dnmt3b* +5mM DCA vs +dH2O

	2	0.380856181	*Dnmt3b* +10mM DCA vs +dH2O
	2	0.289197505	*Dnmt3b* +20mM DCA vs +dH2O
	2	0.407643535	*Tfcp2l1* +5mM DCA vs +dH2O
	2	0.228754887	*Tfcp2l1* +10mM DCA vs +dH2O
	2	0.024638338	*Tfcp2l1* +20mM DCA vs +dH2O
	2	0.028868254	*Fgf5* +5mM DCA vs +dH2O
	2	0.07393582	*Fgf5* +10mM DCA vs +dH2O
	2	0.200097509	*Fgf5* +20mM DCA vs +dH2O

**EV2G**	2	0.131653947	+5mM DCA vs +dH2O
	2	0.120140876	+10mM DCA vs +dH2O
	2	0.152246216	+20mM DCA vs +dH2O

**EV3B**	3	0.304305337	+1mM dm-αKG vs +DMSO
	3	0.008059951	+2mM dm-αKG vs +DMSO
	3	0.00108903	+4mM dm-αKG vs +DMSO

**EV3H**	2	0.331783672	+4mM dm-αKG -> released Activin/Fgf vs 48h EpiLCs
	2	0.051728687	+4mM dm-αKG -> released2i/Lif vs 48h EpiLCs

**EV4A**	4	0.005818559	+4mM Na-Citrate vs +dH2O
	2	0.128714471	+4mM dm-Succinate vs +DMSO

**EV4C**	2	0.045883241	+4mM Na-Citrate vs +dH2O
	2	0.470474746	+4mM dm-Succinate vs+DMSO

**EV4E**	2	0.457979591	+0.1uM CDK4i vs +DMSO
	2	0.433421311	+0.25uM CDK4i vs +DMSO
	2	0.192636681	+0.5uM CDK4i vs +DMSO
	2	0.109439671	+0.75uM CDK4i vs +DMSO
	2	0.037459896	+1uM CDK4i vs +DMSO

**EV4G**	2	0.017500278	*Lin28b* +4mM dm-αKG vs +1uM CDK4i
	2	0.02961682	*Dnmt3b* +4mM dm-αKG vs +1uM CDK4i
	2	0.00112543	*EsrrB* +4mM dm-αKG vs +1uM CDK4i
	2	0.00180433	*Fgf5* +4mM dm-αKG vs +1uM CDK4i
	2	0.02305842	*Klf4* +4mM dm-αKG vs +1uM CDK4i
	2	0.064461172	*Tfcp2l1* +4mM dm-αKG vs +1uM CDK4i

**EV4H**	2	0.061701595	*siKdm3a* vs siControl
	2	0.0327255	*siKdm3b* vs siControl

**EV4I**	2	0.043779467	+dm-αKG vs +DMSO

**EV4J**	5	0.038847101	*Tfcp2l1* (*Tet1/2* DKO vs WT)
	5	0.119118313	*Klf4* (*Tet1/2 DKO vs WT)*

	5	0.481939279	*EsrrB (Tet1/2 DKO vs WT)*
	5	0.008729113	*Lin28b* (*Tet1/2* DKO vs WT)
	5	0.043058337	*Dnmt3b* (*Tet1/2* DKO vs WT)
	5	0.025826395	*Otx2* (*Tet1/*2 DKO vs WT)

**EV5A**	3	0.005722295	*Idh2* (*Prdm1*-GFP+ vs *Prdm1*-GFP-)
	3	0.011751869	*Cox7a1* (*Prdm1*-GFP+ vs *Prdm1*-GFP-)
	3	0.371416263	*Cpt1a (Prdm1-GFP+ vs Prdm1-GFP-)*
	3	0.014510348	*Pdk1 (Prdm1-GFP+ vs Prdm1-GFP-)*
	3	0.070921025	*Pdk3 (Prdm1-GFP+ vs Prdm1-GFP-)*
	3	0.092846755	*Gapdh (Prdm1-GFP+ vs Prdm1-GFP-)*

**EV5C**	2	0.119499001	*Blimp1* AllCytokines+dm-αKG_GFP+ vs AllCytokines_GFP+
	2	0.00320236	*Blimp1* AllCytokines_GFP+ vs AllCytokines_GFP-
	2	0.005772912	*Blimp1* AllCytokines+dm-αKG_GFP+ vsAllCytokines+dm-αKG_GFP-
	2	0.009789637	*Blimp1* AllCytokines+dm-αKG_GFP+ vs AllCytokines_GFP-
	2	0.425403723	*Blimp1* AllCytokines+dm-αKG_GFP- vs AllCytokines_GFP-
	2	0.02205144	*Blimp1* ESCs2iLIF5%FCS vs AllCytokines_GFP-
	2	0.296335044	*Prdm14* AllCytokines+dm-αKG_GFP+ vs AllCytokines_GFP+
	2	0.03368512	*Prdm14* AllCytokines_GFP+ vs AllCytokines_GFP-
	2	0.013636665	*Prdm14* AllCytokines+dm-αKG_GFP+ vsAllCytokines+dm-αKG_GFP-
	2	0.04803251	*Prdm14* AllCytokines+dm-αKG_GFP+ vs AllCytokines_GFP-
	2	0.120338088	*Prdm14* AllCytokines+dm-αKG_GFP- vs AllCytokines_GFP-
	2	0.064752689	*Prdm14* ESCs2iLIF5%FCS vs AllCytokines_GFP-
	2	0.46414875	*Tfap2c* AllCytokines+dm-αKG_GFP+ vs AllCytokines_GFP+
	2	0.002277604	*Tfap2c* AllCytokines_GFP+ vs AllCytokines_GFP-
	2	0.003123253	*Tfap2c* AllCytokines+dm-αKG_GFP+ vsAllCytokines+dm-αKG_GFP-
	2	0.016631347	*Tfap2c* AllCytokines+dm-αKG_GFP+ vs AllCytokines_GFP-
	2	0.193431608	*Tfap2c* AllCytokines+dm-αKG_GFP- vs AllCytokines_GFP-
	2	0.006653792	*Tfap2c* ESCs2iLIF5%FCS vs AllCytokines_GFP-
	2	0.40721904	*T* AllCytokines+dm-αKG_GFP+ vs AllCytokines_GFP+
	2	0.099425306	*T* AllCytokines_GFP+ vs AllCytokines_GFP-
	2	0.002143067	*T* AllCytokines+dm-αKG_GFP+ vsAllCytokines+dm-αKG_GFP-
	2	0.01305198	*T* AllCytokines+dm-αKG_GFP+ vs AllCytokines_GFP-
	2	0.236767046	*T* AllCytokines+dm-αKG_GFP- vs AllCytokines_GFP-
	2	0.016854381	*T* ESCs2iLIF5%FCS vs AllCytokines_GFP-
	2	0.08122742	*Klf4* AllCytokines+dm-αKG_GFP+ vs AllCytokines_GFP+
	2	0.041596401	*Klf4* AllCytokines_GFP+ vs AllCytokines_GFP-
	2	0.030730599	*Klf4* AllCytokines+dm-αKG_GFP+ vsAllCytokines+dm-αKG_GFP-
	2	0.001855506	*Klf4* AllCytokines+dm-αKG_GFP+ vs AllCytokines_GFP-
	2	0.136728153	*Klf4* AllCytokines+dm-αKG_GFP- vs AllCytokines_GFP-
	2	0.004288351	*Klf4* ESCs2iLIF5%FCS vs AllCytokines_GFP-
	2	0.052996385	*Tet1* AllCytokines+dm-αKG_GFP+ vs AllCytokines_GFP+
	2	0.010139781	*Tet1* AllCytokines_GFP+ vs AllCytokines_GFP-
	2	0.000211324	*Tet1* AllCytokines+dm-αKG_GFP+ vsAllCytokines+dm-αKG_GFP-
	2	0.005476443	*Tet1* AllCytokines+dm-αKG_GFP+ vs AllCytokines_GFP-

	2	0.017402671	*Tet1* AllCytokines+dm-αKG_GFP- vs AllCytokines_GFP-
	2	0.063683852	*Tet1* ESCs2iLIF5%FCS vs AllCytokines_GFP-
	2	0.221055583	*Kdm3a* AllCytokines+dm-αKG_GFP+ vs AllCytokines_GFP+
	2	0.012836459	*Kdm3a* AllCytokines_GFP+ vs AllCytokines_GFP-
	2	0.007308207	*Kdm3a* AllCytokines+dm-αKG_GFP+ vsAllCytokines+dm-αKG_GFP-
	2	0.02863749	*Kdm3a* AllCytokines+dm-αKG_GFP+ vs AllCytokines_GFP-
	2	0.257117482	*Kdm3a* AllCytokines+dm-αKG_GFP- vs AllCytokines_GFP-
	2	0.02899936	*Kdm3a* ESCs2iLIF5%FCS vs AllCytokines_GFP-
	2	0.175901061	*Kdm3b* AllCytokines+dm-αKG_GFP+ vs AllCytokines_GFP+
	2	0.023258539	*Kdm3b* AllCytokines_GFP+ vs AllCytokines_GFP-
	2	0.001837072	*Kdm3b* AllCytokines+dm-αKG_GFP+ vsAllCytokines+dm-αKG_GFP-
	2	0.010703307	*Kdm3b* AllCytokines+dm-αKG_GFP+ vs AllCytokines_GFP-
	2	0.09738762	*Kdm3b* AllCytokines+dm-αKG_GFP- vs AllCytokines_GFP-
	2	0.012167613	*Kdm3b* ESCs2iLIF5%FCS vs AllCytokines_GFP-
	2	0.103266633	*Gata6* AllCytokines+dm-αKG_GFP+ vs AllCytokines_GFP+
	2	0.041546603	*Gata6* AllCytokines_GFP+ vs AllCytokines_GFP-
	2	0.092171079	*Gata6* AllCytokines+dm-αKG_GFP+ vsAllCytokines+dm-αKG_GFP-
	2	0.011171875	*Gata6* AllCytokines+dm-αKG_GFP+ vs AllCytokines_GFP-
	2	0.117562607	*Gata6* AllCytokines+dm-αKG_GFP- vs AllCytokines_GFP-
	2	0.0296814	*Gata6* ESCs2iLIF5%FCS vs AllCytokines_GFP-
**EV5F**	2	0.102009272	+SCF+LIF+EGF+dm-αKG+BMP4i vs +SCF+LIF+EGF+dm-αKG+DMSO
	2	0.196881478	+SCF+LIF+EGF+dm-αKG+BMP4i vs +SCF+LIF+EGF+DMSO+DMSO
	2	0.044832839	+SCF+LIF+EGF+dm-αKG+DMSO vs +SCF+LIF+EGF+DMSO+DMSO
**EV6A**	3	0.003469055	+dm-αKG vs +DMSO
**EV6D**	3	0.001602473	*Nanog* enh H3K9me2 ESCs2iLIF vs 48hEpiLCs +DMSO
	3	0.371255077	*Nanog* enh H3K27me3 ESCs2iLIF vs 48hEpiLCs +DMSO
	3	0.006895074	*Nanog* enh H3K9me2 48hEpiLCs +4mM dm-αKG vs 48hEpiLCs +DMSO
	3	0.024282058	*Nanog* enh H3K27me3 48hEpiLCs +4mM dm-αKG vs 48hEpiLCs +DMSO
	3	0.002797141	*Prdm14* enh H3K9me2 ESCs2iLIF vs 48hEpiLCs +DMSO
	3	0.306086379	*Prdm14* enh H3K27me3 ESCs2iLIF vs 48hEpiLCs +DMSO
	3	0.013831917	*Prdm14* enh H3K9me2 48hEpiLCs +4mM dm-αKG vs 48hEpiLCs +DMSO
	3	0.217419344	*Prdm14* enh H3K27me3 48hEpiLCs +4mM dm-αKG vs 48hEpiLCs +DMSO
	3	0.000610051	*Pdgfa* enh H3K9me2 ESCs2iLIF vs 48hEpiLCs +DMSO
	3	7.22861E-05	*Pdgfa* enh H3K27me3 ESCs2iLIF vs 48hEpiLCs +DMSO
	3	0.054135327	*Pdgfa* enh H3K9me2 48hEpiLCs +4mM dm-αKG vs 48hEpiLCs +DMSO
	3	0.011404521	*Pdgfa* enh H3K27me3 48hEpiLCs +4mM dm-αKG vs 48hEpiLCs +DMSO
	3	0.298381604	*Tfap2c* pro H3K9me2 ESCs2iLIF vs 48hEpiLCs +DMSO
	3	0.000155902	*Tfap2c pro* H3K27me3 ESCs2iLIF vs 48hEpiLCs +DMSO
	3	0.128866349	*Tfap2c pro* H3K9me2 48hEpiLCs +4mM dm-αKG vs 48hEpiLCs +DMSO
	3	0.207415776	*Tfap2c pro* H3K27me3 48hEpiLCs +4mM dm-αKG vs 48hEpiLCs +DMSO

Appendix Table S3. P-Values Table.

P-values for all statistically evaluated data sets are listed. For each data set, the corresponding figure panel (column A), the number of independent biological replicates (column B), the *p*-values (column C), and the conditions that were compared (column D) are provided.
