# Supplementary material for: Metabolic regulation of pluripotency and germ cell fate through α‐ketoglutarate
Source: EMBO J. 2018 Sep 26;38(1):e99518. doi: 10.15252/embj.201899518 (PMC6315289; doi:10.15252/embj.201899518)
Supplement: Supplementary file 2 — Expanded View Figures PDF [file EMBJ-38-e99518-s002.pdf]

Expanded View Figures

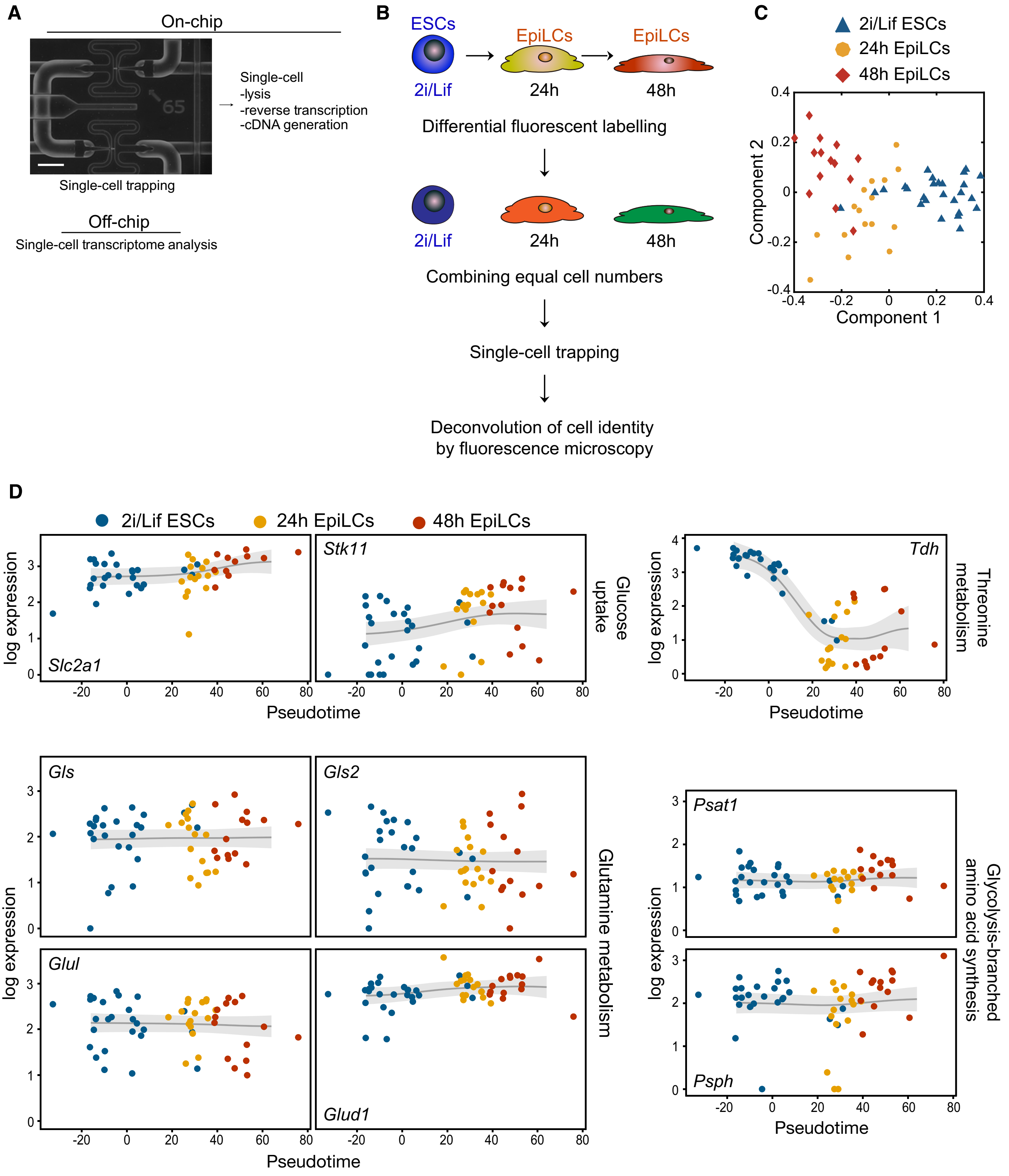

Figure EV1

 © 2018 The Authors

Figure EV1. Single-cell RNA-seq during the ESC-to-EpiLC transition (related to Fig 1).

A Overview of the process flow of single-cell transcriptome analysis using the C1 Single-Cell AutoPrep System (Fluidigm). A magnified image of a microfluidic chip with single cells trapped within individual capture sites is shown. Scale bar, 20 μm.

B Scheme for the concurrent processing of single cells harvested at time points t = 0 h (ESCs 2i/Lif/KSR), t = 24 and t = 48 h following staggered EpiLC induction.

C GPLVM plot of single-cell transcriptome data from ESCs in 2i/Lif/KSR, 24 and 48 h EpiLCs.

D Pseudotime expression trajectories for regulators with key functions in glucose uptake, threonine and glutamine metabolism, and glycolysis-branched amino acid synthesis.

Figure EV2. Glycolytic inhibition sustains a naïve pluripotent state (related to Fig 2).

A–G Examining the effect of glycolytic inhibition on pluripotent state through supplementation of 2-deoxy-D-glucose (2-DG; A–C) and pyruvate dehydrogenase kinase (PDK) inhibitor dichloroacetate (DCA; D–G), respectively, during the ESC-to-EpiLC transition. (A) Western blot showing protein levels for the αKG-generating enzyme IDH2 as a marker for TCA cycle activity after addition of 2-DG and dH_2O, respectively, from t = 48 to t = 72 h during EpiLC differentiation. H3 is used as a loading control. Quantifications are based on two independent experiments. Error bars signify ± SE. *$P \leq 0.05$ (unpaired 1-tailed Student's t-test, see Appendix Table S3 for precise P-values). (B, D) Flow cytometry-based quantification of *Rex1*-GFPd2 cells following 2-DG and DCA supplementation, respectively. (D) Proportions of *Rex1*-GFPd2-positive (GFP+) cells represent averages from duplicate experiments, with error bars denoting ± SE. *P = 0.0403 (unpaired 1-tailed Student's t-test). (C, E) Characteristic bright-field images after addition of 2-DG and DCA, respectively, during the 48 h EpiLC induction. Scale bar, 10 μm. (F) Expression analysis by qRT–PCR of naïve pluripotency regulators and epiblast marker genes in bulk 48 h cells following culture in EpiLC-inducing conditions in the presence of increasing doses of DCA. Graphs show average fold changes in expression over control culture conditions from duplicate experiments. Error bars indicate ± SE. *P = 0.0246 (unpaired 1-tailed Student's t-test). (G) Colony-forming ability subsequent to 48 h DCA treatment. Representative images of AP-stained colonies are displayed. Scale bar, 250 μm. Colony formation following DCA supplementation is normalized to control-treated cells. Graphs represent averages from duplicate experiments. Error bars signify ± SE.

© 2018 The Authors

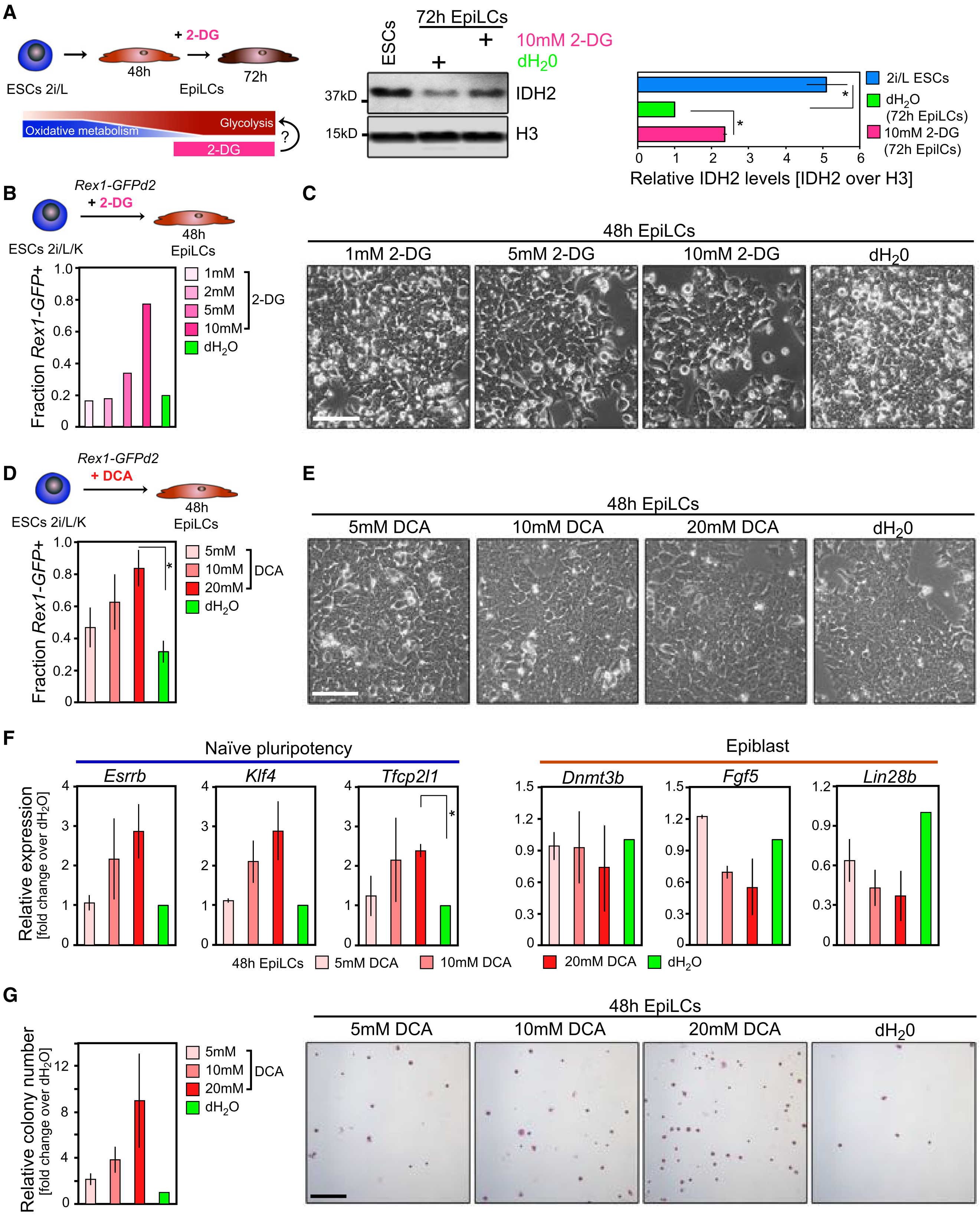

Figure EV2.

 © 2018 The Authors

Figure EV3. The effect of αKG on naïve pluripotency is concentration-dependent and fully reversible (related to Fig 3).

A Representative Western blot showing DNMT3b, IDH2, H3K27me3 and H3K27me2 dynamics during the ESC-to EpiLC transition. H3 is used as a loading control.

B–D Investigating the impact of dm-αKG supplementation during the ESC-to-EpiLC transition on pluripotent state. (B) Flow cytometer analysis of *Rex1*-GFPd2-positive (*Rex1*-GFP+) cells. Graphs represent average fractions of *Rex1*-GFP+ cells quantified from triplicate experiments. Error bars denote ± SE. $**P \leq 0.01$; $***P \leq 0.005$ (unpaired 1-tailed Student's *t*-test, see Appendix Table S3 for precise *P*-values). (C) Characteristic bright-field images of *Rex1*-GFPd2 cells following 48 h culture in EpiLC-inducing conditions in the presence of increasing doses of dm-αKG. Scale bar, 10 μm. (D) Representative Western blot displaying IDH2 levels following 48 h EpiLC stimulation with 4 mM dm-αKG and DMSO, respectively. H3 is used as a loading control.

E Expression analysis by qRT–PCR of key regulators of naïve pluripotency and epiblast genes in bulk 48 h cells. Transcript levels are normalized to levels in control culture conditions.

F Colony formation after culture with increasing dm-αKG concentration during the ESC-to-EpiLC transition, normalized to control culture conditions. Representative images of AP-stained colonies are displayed. Scale bar, 250 μm.

G Super-resolution images of TOM-20 immune-stained outer mitochondrial membranes (in magenta) following 48 h culture in 2i/Lif/KSR and EpiLC-inducing conditions with 4 mM dm-αKG and DMSO, respectively. Cell membranes (in green) are stained with Alexa-488-coupled wheat germ agglutinin (WGA-488). Scale bars, 10 and 3 μm (in zoomed-in images), respectively.

H Flow cytometry-based quantification of *Rex1*-GFPd2-positive (*Rex1*-GFP+) cells following 4 mM dm-αKG supplementation during the ESC-to-EpiLC transition, and subsequent release into standard 2i/Lif/KSR and EpiLC culture conditions, respectively. Average fractions of *Rex1*-GFP+ cells are calculated from duplicate experiments each. Error bars indicate ± SE. $*P = 0.0517$ (unpaired 1-tailed Student's *t*-test).

I Flow cytometer analysis of *Rex1*-GFPd2-positive (GFP+) cells following 48 h dm-αKG pre-treatment of ESCs in 2i/Lif/KSR culture conditions and subsequent release into EpiLC-inducing culture conditions.

© 2018 The Authors

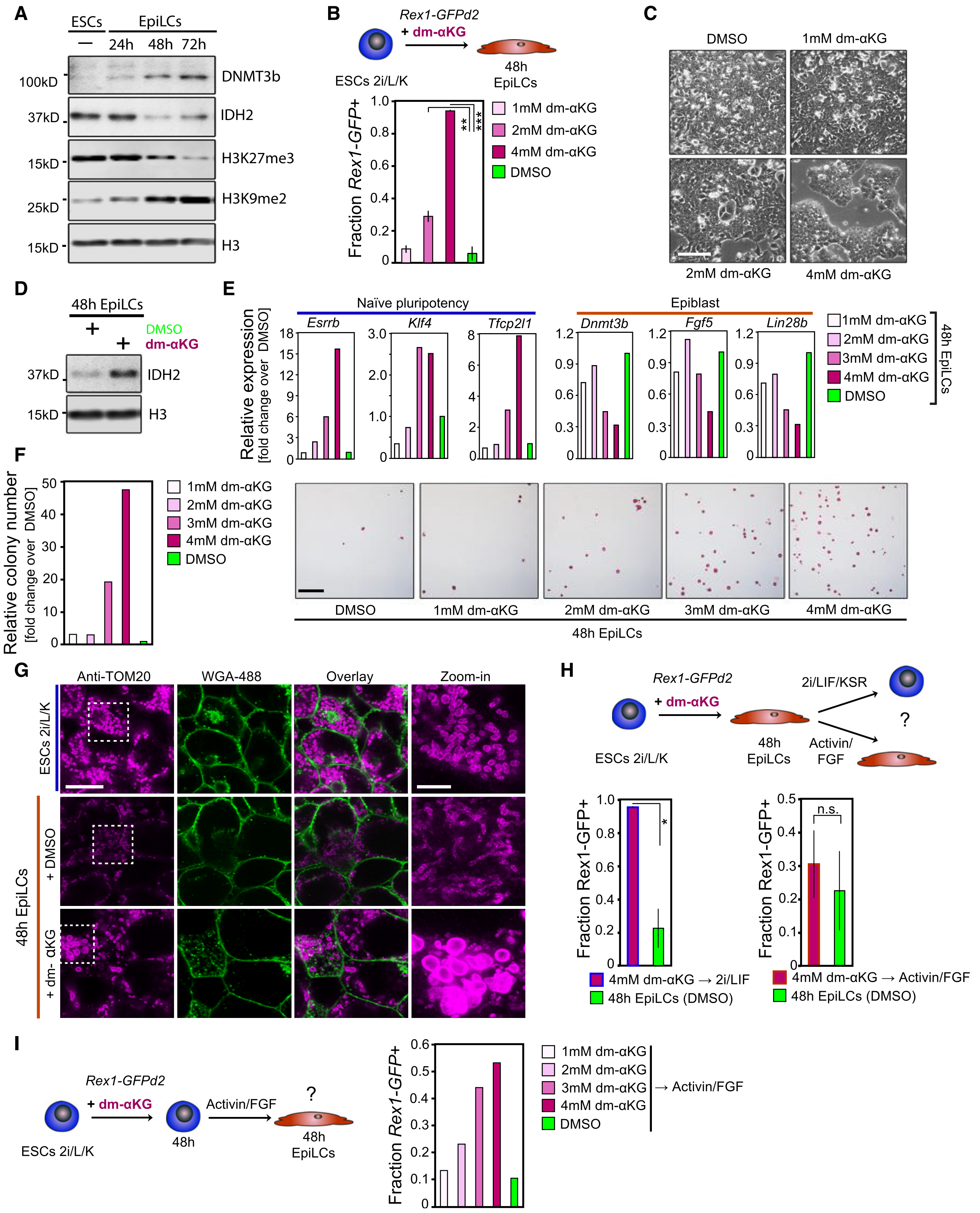

Figure EV3.

© 2018 The Authors

Figure EV4. The effect of αKG on naïve pluripotency is specific and mediated via cell cycle and epigenetic effects (related to Fig 3).

A Flow cytometer analysis of *Rex1*-GFPd2 cells following 48 h EpiLC stimulation in the presence of 4 mM sodium citrate dehydrate (Na-citrate; graphs represent averages from quadruplicate assays) and 4 mM dimethyl succinate (dm-succinate; graphs show averages from duplicate assays). Error bars signify ± SE. **P = 0.0058 (unpaired 1-tailed Student's t-test).

B Representative bright-field images after 48 h EpiLC induction with 4 mM Na-citrate and dm-succinate, respectively. Scale bar, 10 μm.

C Colony-forming ability following addition of 4 mM Na-citrate and dm-succinate, respectively, during the EpiLC induction. Graphs represent colony formation normalized to control culture conditions, averaged over duplicate experiments each. Error bars denote ± SE. *P = 0.0459 (unpaired 1-tailed Student's t-test).

D, E Investigating the impact of proliferation rate on cell state. (D) Cell proliferation analysis through CellTrace Violet labelling of *Rex1*-GFPd2 ESCs in 2i/Lif/KSR culture conditions (t = 0 h) followed by flow cytometry-based evaluation of dye dilution in the presence of 4 mM dm-αKG and DMSO, respectively, at t = 72 h. (E) Flow cytometer-based quantification of *Rex1*-GFPd2 cells following EpiLC stimulation in the presence of increasing concentrations of CDK4 inhibitor (CDK4i). Graphs represent the average fractions of *Rex1*-GFPd2-positive (*Rex1*-GFP+) cells from duplicate experiments. Error bars indicate ± SE. *P = 0.0375 (unpaired 1-tailed Student's t-test).

F FACS profiles of CellTrace Violet-labelled *Rex1*-GFPd2 cells at t = 48 h, following supplementation of 4 mM dm-αKG and 1 μM CDK4i, respectively, during the ESC-to-EpiLC transition. Cells are gated based on CellTrace Violet intensities (+++, CellTrace Violet-high; +, CellTrace Violet-low) and collected for transcript profiling (see G).

G qRT–PCR analysis of FACS-sorted CellTrace Violet-labelled *Rex1*-GFPd2 cells of matching CellTrace Violet intensities. Transcript levels are normalized to levels in CDK4i-treated cells, averaged over both CellTrace Violet-high (+++) and CellTrace Violet-low (+) fractions, and represent duplicate experiments. Error bars denote ± SE. *$P \leq 0.05$; ***$P \leq 0.005$ (unpaired 1-tailed Student's t-test, see Appendix Table S3 for all P-values).

H Expression analysis by qRT–PCR confirms the small-interfering RNA (siRNA)-mediated knockdown of *Kdm3a* and *Kdm3b* in ESCs in 2i/Lif conditions. Knockdown efficiencies represent expression levels at t = 48 h after siRNA transfection normalized to levels prior to siRNA transfection (t = 0 h) and are averaged over duplicate experiments. Error bars signify ± SE. *P = 0.0327 (unpaired 1-tailed Student's t-test).

I Colony-forming abilities of cells subsequent to the combinatorial knockdown of *Kdm3a* and *Kdm3b* and EpiLC differentiation in the presence of 4 mM dm-αKG and DMSO, respectively. Representative images of AP-positive colonies are displayed. Scale bar, 250 μm. Graphs show relative colony formation following *Kdm3a/b* knockdown, normalized to non-targeting control siRNA-treated cells derived under identical culture conditions, averaged over duplicate assays. Error bars denote ± SE. *P = 0.0438 (unpaired 1-tailed Student's t-test).

J Expression analysis by qRT–PCR of naïve pluripotency and epiblast marker genes in *Tet1/2* wild-type and double-knockout (DKO) cells following 4 mM dm-αKG and DMSO, respectively, supplementation during the 48 h EpiLC induction. Transcript levels are normalized to levels in the respective control-treated cells. Averages of five independent biological assays are shown. Error bars indicate ± SE. *$P \leq 0.05$; **$P \leq 0.01$ (unpaired 1-tailed Student's t-test, see Appendix Table S3 for all P-values).

© 2018 The Authors

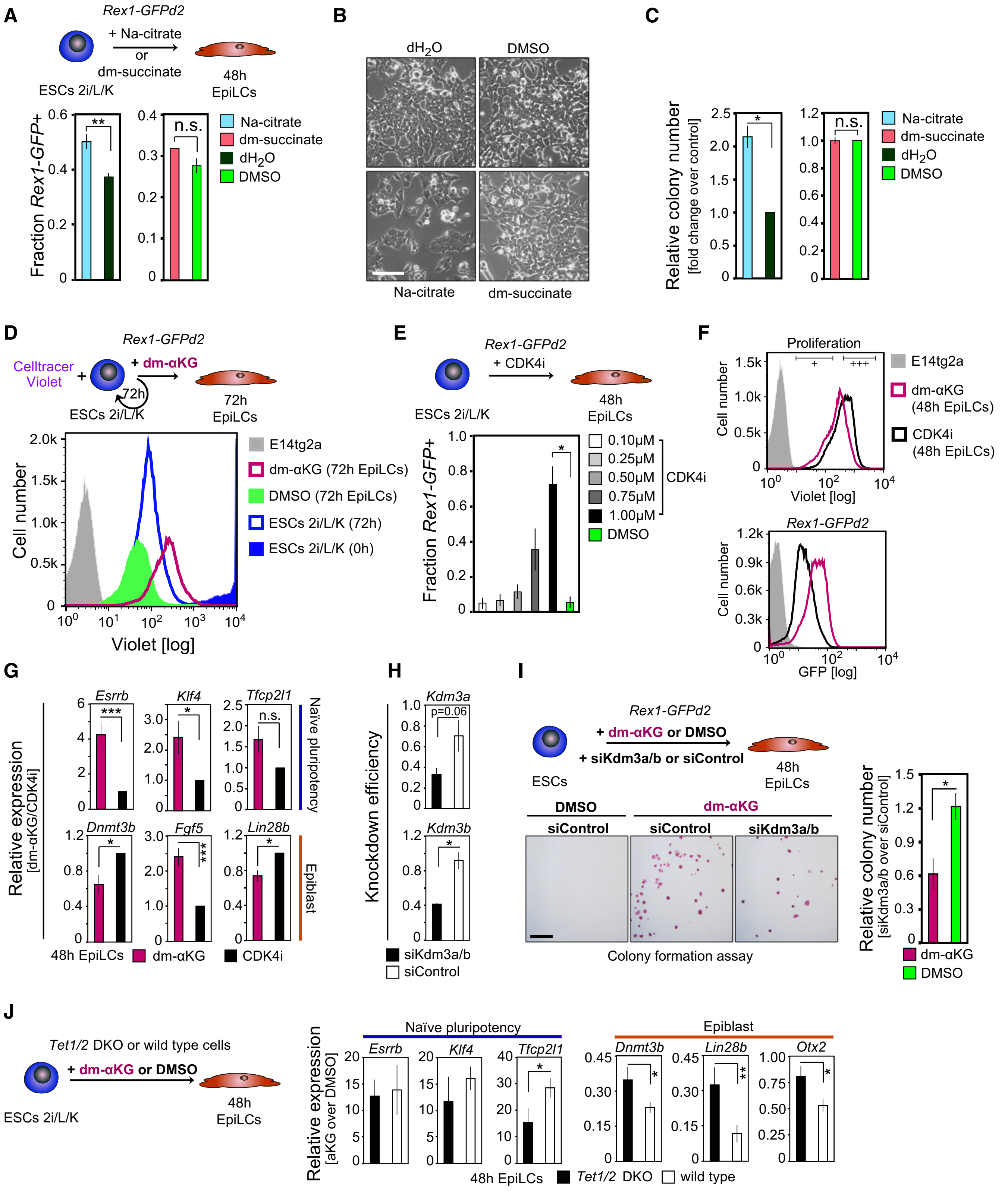

Figure EV4.

© 2018 The Authors

Figure EV5. αKG promotes PGC fate (related to Fig 4).

A qRT–PCR analysis of key regulators implicated in oxidative (*Cox7a1*, *Cpt1a*), glycolytic (*Pdk1*, *Pdk3*, *Gapdh*) and αKG (*Idh2*) metabolism, respectively, in FACS-purified *Prdm1*-GFP-positive (GFP+) and *Prdm1*-GFP-negative (GFP−) cells of day-4 PGCLC embryoids. Transcript levels are normalized to levels in GFP− fractions of control-treated embryoids. Graphs represent triplicate experiments. Error bars indicate ± SE. *$P \leq 0.05$; **$P \leq 0.01$ (unpaired 1-tailed Student's *t*-test, see Appendix Table S3 for all *P*-values).

B, C PGCLC induction in the presence of PGC cytokines and 4 mM dm-αKG. (B) Characteristic bright-field and corresponding fluorescence images of day-4 *Prdm1*-GFP PGCLC aggregates are shown. Scale bar, 20 μm. Fluorescent image intensity scales (in units of counts) are adjusted equally. (C) Expression analysis by qRT–PCR in FACS-purified *Prdm1*-GFP-positive (GFP+) and *Prdm1*-GFP-negative (GFP−) cells at day 4 of PGCLC induction. Transcript levels are normalized to levels in GFP− fractions of control-treated embryoids. Graphs represent duplicate experiments. Error bars indicate ± SE. *$P \leq 0.05$; **$P \leq 0.01$; ***$P \leq 0.005$ (unpaired 1-tailed Student's *t*-test, see Appendix Table S3 for all *P*-values).

D, E PGCLC differentiation upon addition of 4 mM dm-αKG, without cytokines. (D) Characteristic bright-field and fluorescence images of *Prdm1*-GFP embryoids after 2 days of aggregation with LIF (10 ng ml^{-1}) and BMP4 (500 ng ml^{-1}) or dm-αKG (4 mM) are presented. Scale bar, 20 μm. Fluorescent image intensity scales (in units of counts) are adjusted equally. (E) Expression analysis by qRT–PCR of the key PGC regulators *Prdm1*, *Prdm14* and *Tfap2c* in FACS-purified *Prdm1*-GFP-positive (GFP+) and *Prdm1*-GFP-negative (GFP−) embryoids following 2 days of aggregation in the presence of LIF (10 ng ml^{-1}) and BMP4 (500 ng ml^{-1}) or dm-αKG (4 mM). Transcript levels are normalized to levels in GFP− cells from BMP4-stimulated embryoids.

F Flow cytometer-based quantification of *Prdm1*-GFP-positive (GFP+) cells following 4 days of aggregation in the presence of 4 mM dm-αKG and 500 nM inhibitor of BMP signalling (iBMP), without external BMP4/8 supplementation. Average fractions of GFP+ cells are quantified from duplicate assays. Error bars denote ± SE.

© 2018 The Authors

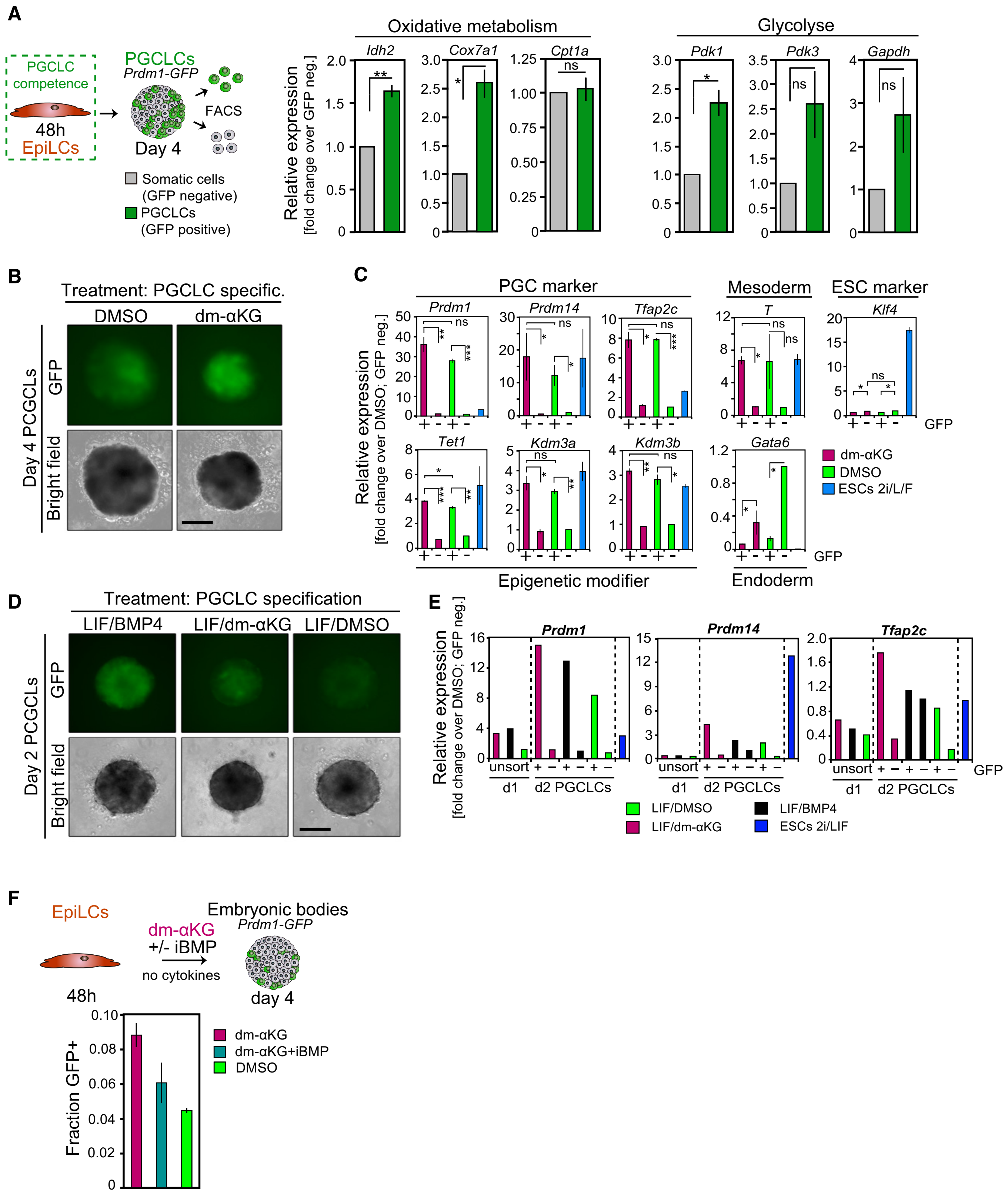

Figure EV5.

 © 2018 The Authors

Figure EV6. αKG prolongs the developmental competence for the PGC fate (related to Fig 4).

A, B Day-4 *Prdm1*-GFP aggregates induced from 48 h EpiLCs following dm-αKG supplementation from t = 24 to t = 48 h during the EpiLC differentiation. (A) Characteristic flow cytometer profiles are presented. Average proportions of *Prdm1*-GFP-positive (GFP+) cells are quantified from triplicate experiments. Error bars indicate ± SE. ****P* = 0.0035 (unpaired 1-tailed Student's *t*-test). (B) Representative bright-field and fluorescent images of day-4 *Prdm1*-GFP aggregates are shown. Scale bar, 20 μm. Fluorescent image intensity scales (in units of counts) are adjusted equally.

C Characteristic bright-field and fluorescent images of day-4 *Prdm1*-GFP aggregates specified from 72 h EpiLCs following dm-αKG supplementation from t = 48 to t = 72 h during the EpiLC differentiation are presented. Scale bar, 20 μm. Fluorescent image intensity scales (in units of counts) are adjusted equally.

D ChIP-qPCR analysis of H3K9me2 and H3K27me3 in selected cis-regulatory regions in naïve ESCs and at t = 48 h following EpiLC induction in the presence of 4 mM dm-αKG and DMSO, respectively. Graphs show enrichment of H3K9me2, H3K27me3 and IgG control, respectively, relative to DMSO-treated EpiLCs. Averages represent triplicate independent experiments. Error bars indicate ± SE. **P* ≤ 0.05; ***P* ≤ 0.01; ****P* ≤ 0.005 (unpaired 1-tailed Student's *t*-test, see Appendix Table S3 for all *P*-values).

E Model illustrating the effect of αKG on the epigenetic state and, in turn, cell fate competency.

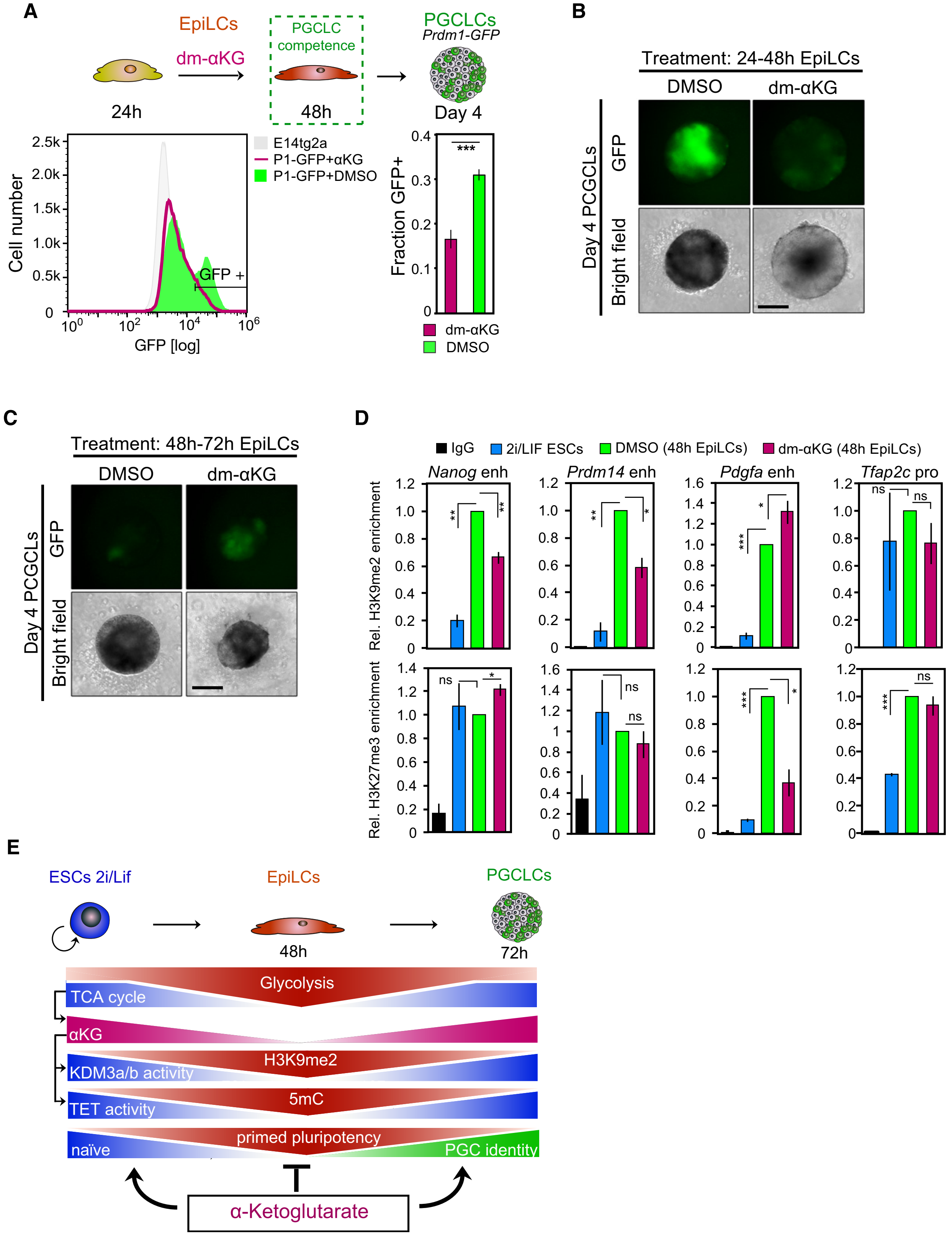

Figure EV6.

© 2018 The Authors
